# A Comparative Study of Human Thermal Face Recognition Based on Haar Wavelet Transform and Local Binary Pattern

**DOI:** 10.1155/2012/261089

**Published:** 2012-07-04

**Authors:** Debotosh Bhattacharjee, Ayan Seal, Suranjan Ganguly, Mita Nasipuri, Dipak Kumar Basu

**Affiliations:** Department of Computer Science and Engineering, Jadavpur University, Kolkata 700032, India

## Abstract

Thermal infrared (IR) images focus on changes of temperature distribution on facial muscles and blood vessels. These temperature changes can be regarded as texture features of images. A comparative study of face two recognition methods working in thermal spectrum is carried out in this paper. In the first approach, the training images and the test images are processed with Haar wavelet transform and the LL band and the average of LH/HL/HH bands subimages are created for each face image. Then a total confidence matrix is formed for each face image by taking a weighted sum of the corresponding pixel values of the LL band and average band. For LBP feature extraction, each of the face images in training and test datasets is divided into 161 numbers of subimages, each of size 8 × 8 pixels. For each such subimages, LBP features are extracted which are concatenated in manner. PCA is performed separately on the individual feature set for dimensionality reduction. Finally, two different classifiers namely multilayer feed forward neural network and minimum distance classifier are used to classify face images. The experiments have been performed on the database created at our own laboratory and Terravic Facial IR Database.

## 1. Introduction

In the modern society, there is an increasing need to track and recognize persons automatically in various areas such as in the areas of surveillance, closed circuit television (CCTV) control, user authentication, human computer interface (HCI), daily attendance register, airport security checks, and immigration checks [1–3]. Such requirement for reliable personal identification in computerized access control has resulted in an increased interest in biometrics. The key element of biometric technology is its ability to identify a human being and enforce security. Nearly all-biometric systems work in the same manner. First, a person is registered into a database using a specified method. Information about a certain characteristic of the human is captured. This information is usually placed through an algorithm that turns the information into a code that the database stores. When the person needs to be identified, the system will take the information about the person again, translate this new information with the algorithm, and then compare the new code with the stored ones in the database to find out a possible match. Biometrics use physical characteristics or personal traits to identify a person. Physical feature is suitable for identity purpose and generally obtained from living human body. Commonly used physical features are fingerprints, facial features, hand geometry, eye features (iris and retina), and so forth. So biometrics involve using the different parts of the body. Personal trait is sometimes more appropriate for some applications which need direct physical interaction. The most commonly used personal traits are signature and voices and so forth. Among many biometric security systems, face recognition has drawn significant attention of the researchers for the last three decades because of its potential applications in security system. There are a number of reasons to choose face recognition for designing efficient biometric security systems. The most important one is that no physical interaction is needed. This is helpful for the cases where touching is prohibited due to hygienic reasons or religious or cultural traditions. Most of the research works in this area have focused on visible spectrum imaging due to easy availability of low cost visible band optical cameras. But it requires an external source of illumination. Even with a considerable success for automatic face recognition techniques in many practical applications, the task of face recognition based only on the visible spectrum is still a challenging problem under uncontrolled environments. The challenges are even more philosophical when one considers the large variations in the visual stimulus due to illumination conditions, poses [4], facial expressions, aging, and disguises such as facial hair, glasses, or cosmetics. Performance of visual face recognition is sensitive to variations in illumination conditions and usually degrades significantly when the lighting is not bright or when it is not illuminating the face uniformly. The changes caused by illumination on the same individual are often larger than the differences between individuals. Various algorithms (e.g., histogram equalization, eigenfaces, etc.) for compensating such variations have been studied with partial success. These techniques try to reduce the within-class variability introduced by changes in illumination. To overcome this limitation, several solutions have been designed. One solution is using 3D data obtained from 3D vision device. Such systems are less dependent on illumination changes, but they have some disadvantages: the cost of such systems is high, and their processing speed is low. Thermal IR images [5] have been suggested as a possible alternative in handling situations where there is no control over illumination. The wavelength ranges of different infrared spectrums are shown in Table 1. 

Thermal IR band is more popular to the researchers working with thermal images. Recently researchers have been using near-IR imaging cameras for face recognition with better results [6], but SWIR and MWIR have not been used significantly till now. Thermal IR images represent the heat patterns emitted from an object, and they do not consider the reflected energy. Objects emit different amounts of IR energy according to their body temperature and characteristics. Previously, thermal IR camera was costly, but recently the cost of such cameras has come down considerably with the development of CCD technology [7]. Thermal images can be captured under different lighting conditions, even under completely dark environment. Using thermal images, the tasks of face detection, localization, and segmentation are comparatively easier and more reliable than those in visible band images [8]. Humans are homoeothermic and hence capable of maintaining constant temperature under different surrounding temperature, and since blood vessels transport warm blood throughout the body, the thermal patterns of faces are derived primarily from the pattern of blood vessels under the skin. The vein and tissue structure of the face is unique for each human being [9], and therefore the IR images are also unique. It is known that even identical twins have different thermal patterns. An infrared camera with good sensitivity can capture images of superficial blood vessels on the human face [10] without any physical interaction. However, it has been indicated by Guyton and Hall [11] that the average diameter of blood vessels is around 10–15 *μ*m, which is too small to be detected by current IR cameras because of the limitation in spatial resolution. The skin just above a blood vessel is on an average 0.1°C warmer than the adjacent skin, which is beyond the thermal accuracy of current IR cameras. However, the convective heat transfer effect from the flow of “hot” arterial blood in superficial vessels creates characteristic thermal imprints, which are at a gradient with the surrounding tissue. Face recognition based on thermal IR spectrum utilizes the anatomical information [12] of human face as features unique to each individual while sacrificing color recognition. Therefore, the infrared image recognition should focus on thermal distribution patterns on the facial muscles and blood vessels, mainly on cheek, forehead, and nasal tip. These regional thermal distribution patterns can be regarded as the texture pattern unique for a particular face. Wavelet transform can be used to detect the multiscale, multidirectional changes of texture. Local binary patterns (LBPs) are also a well-known texture descriptor and also a successful local descriptor for face recognition under local illumination variations. Therefore, this paper describes that a comparative study of different approach of thermal IR human face recognition system is proposed. The paper is organized as follows: Section 2 presents the outline proposed system. In Section 3, the comparative analyses of these methods in the database created at our own laboratory and Terravic Facial IR Database are presented. Finally, in Section 4, results are discussed and conclusions are given.

## 2. Outline of the Proposed System

**Figure 1:**
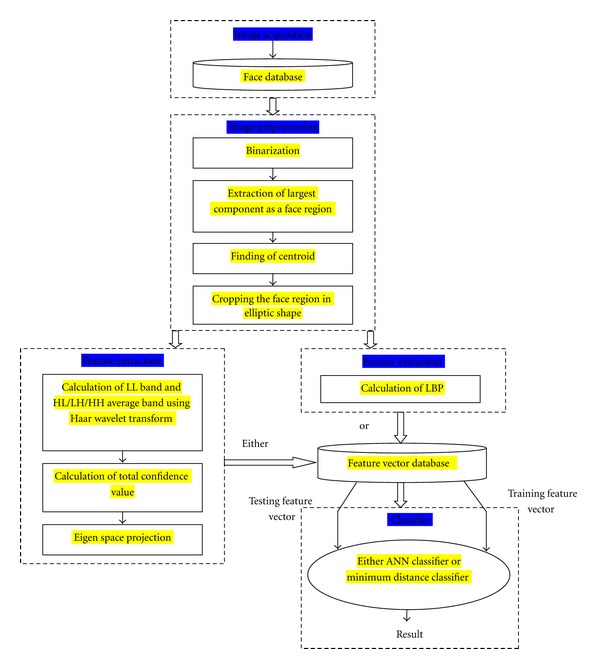
Schematic block diagram of the proposed system.

The proposed thermal face recognition system (TFRS) can be subdivided into four main parts, namely, image acquisition, image preprocessing, feature extraction, and classification. The image preprocessing part involves binarization of the acquired thermal face image, extraction of largest component as the face region, finding the centroid of the face region, and finally cropping of the face region in elliptic shape. The two different features extraction techniques have been discussed in this paper. The first one is to find LL band and HL/LH/HH average band images using Haar wavelet transform, and the total confidence matrix is used as a feature vector. The eigenspace projection is performed on feature vector to reduce the dimensionality. This reduced feature vector is fed into a classifier. The second method of features extraction technique is local binary pattern (LBP). As a classifier, a back propagation feed forward neural network or a minimum distance classifier is used in this paper. The block diagram of the proposed system is given in Figure 1. The system starts with acquisition of thermal face image and end with successful classification. The set of image processing and classification techniques which have been used here is discussed in detail in subsequent subsections. 

### 2.1. Thermal Face Image Acquisition

**Figure 2:**
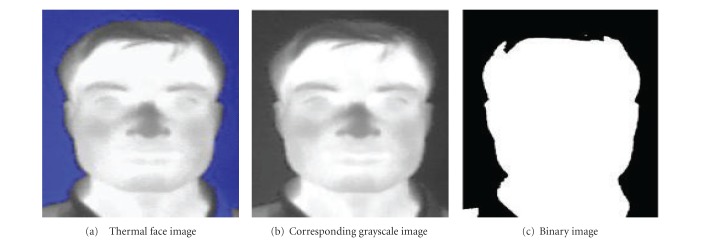
Thermal face image and its various preprocessing stages.

In the present work, unregistered thermal and visible face images are acquired simultaneously with variable expressions, poses, and with/without glasses. Till now 17 individuals have volunteered for this photo shots, and for each individual 34 different templates of RGB color images with different expressions, namely, (Exp1) happy, (Exp2) angry, (Exp3) sad, (Exp4) disgusted, (Exp5) neutral, (Exp6) fearful and (Exp7) surprised are available. Different pose changes about *x*-axis, *y*-axis, and *z*-axis are also available. Resolution of each image is 320 × 240, and the images are saved in 24-bit JPEG format. Two different cameras are used to capture this database. One is thermal—FLIR 7, and another is Optical—Sony cyber shot. A typical thermal face image is shown in Figure 2(a). This thermal face image depicts interesting thermal information of a facial model.

### 2.2. Binarization

The binarization of 24-bit colour image is divided into two steps. In the first step, the colour image is converted into an 8-bit grayscale image using

(1) I=(0.2989×redcomponent)+(0.5870×greencomponent) +(0.1140×  bluecomponent),

where “*I*” is the grayscale image. The grayscale image corresponding to the thermal image of Figure 2(a) is shown in Figure 2(b). Grayscale image is then converted into binary image. For this purpose, mean gray value of grayscale image (say *g*_mean_) is computed with the help of

(2) $$\begin{array}{c} g_{\text{mean}}=\frac{\sum_{i=1}^{\text{row}}\sum_{j=1}^{\text{column}}g\left(i,j\right)}{\left(\text{row}\times \text{column}\right)}. \end{array}$$

If the gray value of any pixel (*i*, *j*) (say *g*(*i*, *j*)) is greater than or equal to *g*_mean_, then the pixel location in the binary image (*i*, *j*) is set with 1 (white), else it is set with 0 (black). The binarization process can be mathematically expressed with the help of

(3) b(i,j)={1if  g(i,j)≥gmean0otherwise.

In a binary image, black pixels mean background and are represented with “0”s, whereas white pixels mean the face region and are represented with “1”s. The binary image corresponding to the grayscale image of Figure 2(b) is shown in Figure 2(c).

### 2.3. Extraction of Largest Component

**Figure 3:**
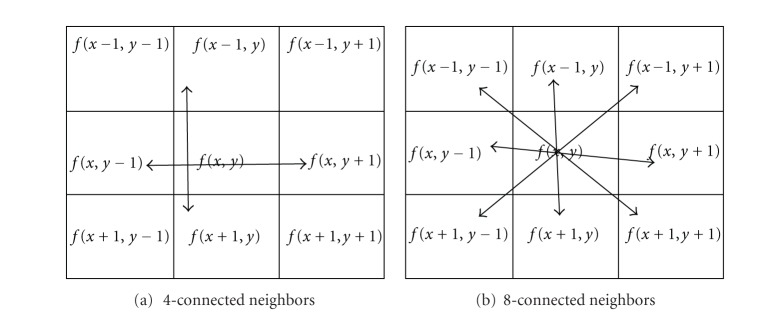
Different connected neighborhoods.

The foreground of a binary image may contain more than one object or components. Say, in Figure 2(c), it has three objects or component. The large one represents the face region. The others are at the left hand bottom corner and a small dot on the top. Then the largest component has been extracted from binary image using “connected component labeling” algorithm [13]. This algorithm is based on either “4-conneted” neighbours or “8-connected” neighbours method [14]. In “4-connected” neighbours method, a pixel is considered as connected if it has neighbours on the same row or column. This is illustrated in Figure 3(a). Suppose the central pixel of a 3 × 3 mask “*f*” is *f*(*x*, *y*), then this method will consider the pixels *f*(*x* + 1, *y*), *f*(*x* − 1, *y*), *f*(*x*, *y* + 1), and *f*(*x*, *y* − 1) for checking the connectivity of *f*(*x*, *y*). In “8-connected” method besides the row and columns neighbours, the diagonal neighbours are also checked. That means “4-connected” pixels plus the diagonal pixels are called an “8-connected” neighbour which is illustrated in Figure 3(b). Thus, for a central *f*(*x*, *y*) of a 3 × 3 mask “*f*” the “8-connected” neighbour methods will consider *f*(*x* − 1, *y* − 1), *f*(*x* − 1, *y*), *f*(*x* − 1, *y* + 1), *f*(*x*, *y* − 1), *f*(*x*, *y* + 1), *f*(*x* + 1, *y* − 1), *f*(*x* + 1, *y*), and *f*(*x* + 1, *y* + 1) for checking the connectivity of *f*(*x*, *y*).

**Figure 4:**
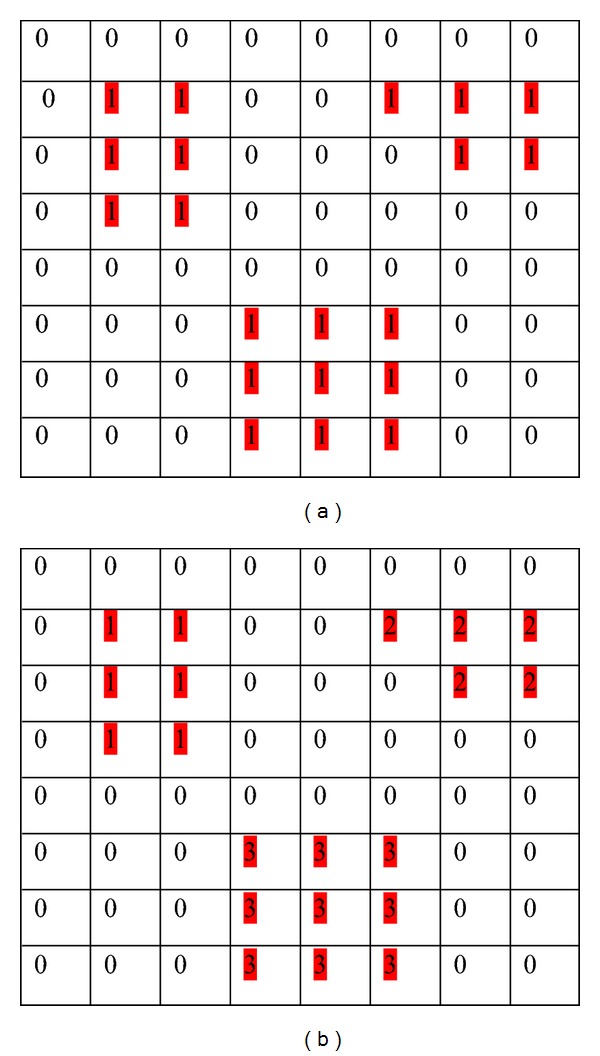
(a) Connected component, (b) Labeled connected components.

A pixel to be connected to itself is called reflexive. A pixel and its neighbour are mutually connected is called symmetric. 4-connectivity and 8-connectivity are also transitive: if A is connected to pixel B, and pixel B is connected to pixel C, then there exists a connected path between pixels A and C. A relation (such as connectivity) is called an equivalence relation if it reflexive, symmetric and transitive. All equivalence classes of connected pixels in a binary image, is called connected component labelling. The result of connected component labelling is another image in which everything in one connected region is labeled “1” (for example), everything in another connected region is labeled “2”, and so forth. For example, the binary image in Figure 4(a) has three connected components and three labeled connected components is shown in Figure 4(b).

“Connected component labeling” algorithm is given in Algorithm 1.

**Figure 5:**
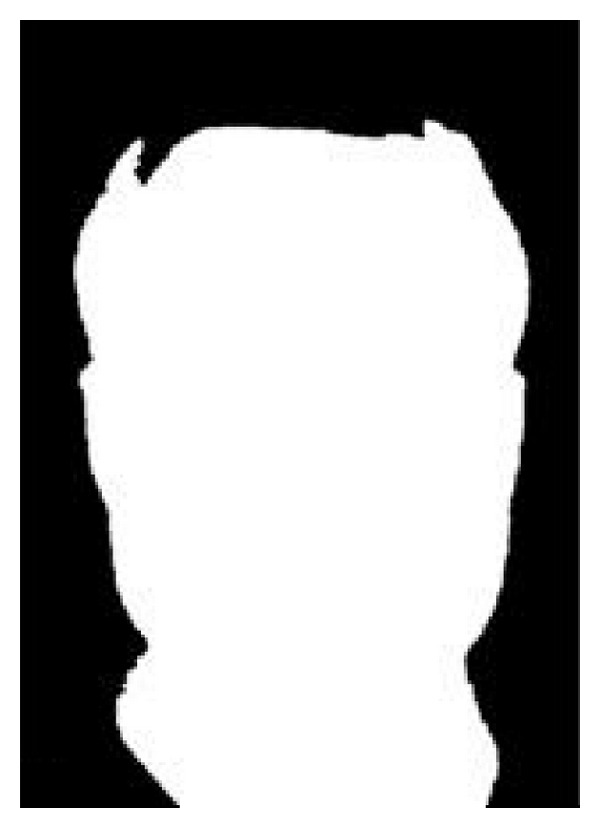
The largest component as a face skin region.

Using “connected component labeling” algorithm, the largest component of face region is identified from Figure 2(c) which is shown in Figure 5.

### 2.4. Finding the Centroid [15]

Centroid has been extracted from the largest component of the binary image using

(4) $$\begin{array}{c} X=\frac{\sum m_{f(x,y)}x}{\sum m_{f(x,y)}},\\ Y=\frac{\sum m_{f(x,y)}y}{\sum m_{f(x,y)}}, \end{array}$$

where *x*, *y* are the coordinate, of the binary image and *m* is the intensity value that is *m*_*f*(*x*,*y*)_ = *f*(*x*, *y*) = 0 or 1.

### 2.5. Cropping of the Face Region in Elliptic Shape

**Figure 6:**
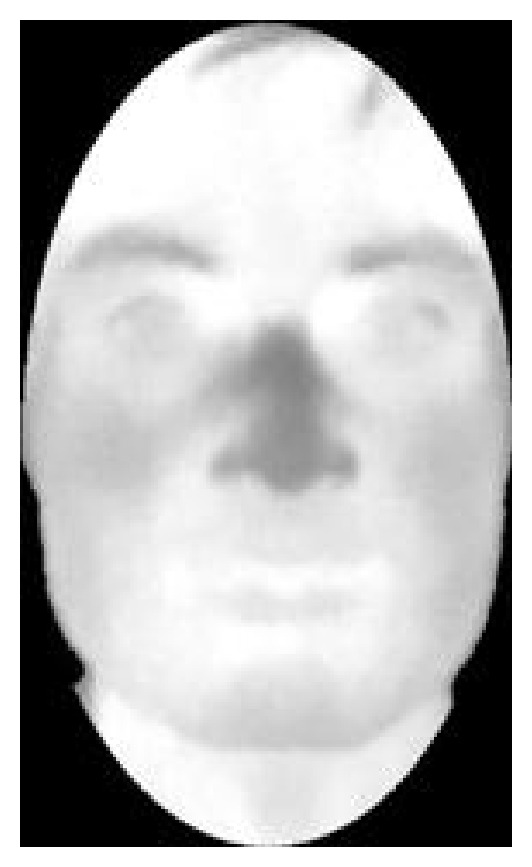
Cropped face region in elliptic shape.

Normally, human face is of ellipse shape. Then, from the above centroid coordinates, human face has been cropped in elliptic shape using “Bresenham ellipse drawing” [16] algorithm. This algorithm takes the distance between the centroid and the right ear as the minor axis of the ellipse and distance between the centroid and the fore head as major axis of the ellipse. The pixels selected by the ellipse drawing algorithm are mapped onto the gray level image of Figure 2(b), and finally the face region is cropped. This is shown in Figure 6.

### 2.6. Calculate LL and HL/LH/HH Average Band Using Haar Wavelet Transform

The first method of feature extraction is discrete wavelet transform (DWT). The DWT was invented by the Hungarian mathematician Alfréd Haar in 1909. A key advantage of wavelet transform over Fourier transforms is temporal resolution. Wavelet transform captures both frequency and spatial information. The DWT has a huge number of applications in science, engineering, computer science, and mathematics. The Haar transformation is used here since it is the simplest wavelet transform of all and can successfully serve our purpose. Wavelet transform has merits of multiresolution, multiscale decomposition and so on. To obtain the standard decomposition [17] of a 2D image, the 1D wavelet transform to each row is applied first. This operation gives an average pixel value along with detail coefficients for each row. These transformed rows are treated as if they were themselves in an image. Now, 1D wavelet transform to each column is applied. The resulting pixel values are all detail coefficients except for a single overall average coefficient. As a result, the elliptical shape facial image is decomposed into four regions that can be gained. These regions are one low-frequency LL_1_ (approximate component), and three high-frequency regions (detailed components), namely, LH_1_ (horizontal component), HL_1_ (vertical component) and HH_1_ (diagonal component), respectively. The low frequency subband LL_1_ can be further decomposed into four subbands LL_2_, LH_2_, HL_2_, and HH_2_ at the next coarse scale. LL_i_ is a reduced resolution corresponding to the low-frequency part of an image. The sketch map of the quadratic wavelet decomposition is shown in Figure 6.

**Figure 7:**
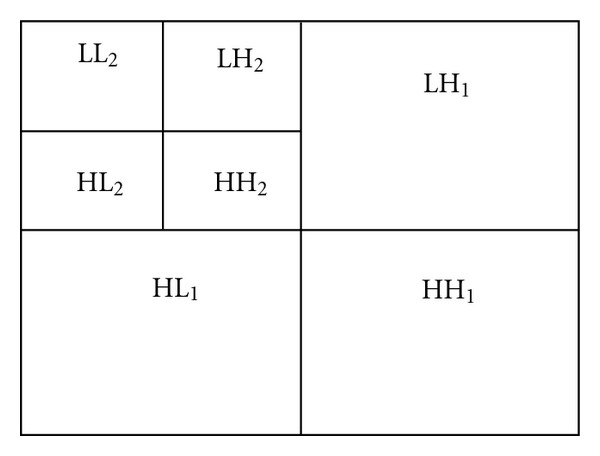
Sketch map of the quadratic wavelet decomposition.

As illustrated in Figure 7, the L denotes low frequency and the H denotes high frequency, and subscripts named from 1 to 2 denote simple, quadratic wavelet decomposition, respectively. The standard decomposition algorithm is given in Algorithm 2.

Let us start with a simple example of 1D wavelet transform [18]. Suppose an image with only one row of four pixels, having intensity values [10 4 9 5]. Now apply the Haar wavelet transform on this image. To do so, first pair up the input intensity values or pixel values, storing the mean in order to get the new lower resolution image with intensity values [7 7]. Obviously, some information might be lost in this averaging process. Some detail coefficients need to be stored to recover the original four intensity values from the two mean values, which capture the missing information. In this example, 3 is the first detail coefficient, since the computed mean is 3 less than 10 and 3 more than 4. This single number is responsible to recover the first two pixels of original four-pixel image. Similarly, the second detail coefficient is 2. Thus, the original image is decomposed into a lower resolution (two-pixel) version and a pair of detail coefficients. Repeating this process recursively on the averages gives the full decomposition, which is shown in Table 2.

**Figure 8:**
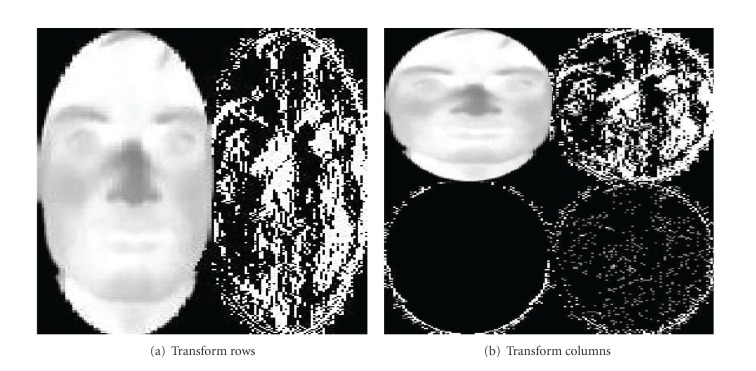
Haar wavelet transform.

Thus, the one-dimensional Haar transform of the original four-pixel image is given by [7  0  3  2]. After applying standard decomposition algorithm on Figure 6, the resultant figure is shown in Figure 8.

The pixels of LL_2_ image can be rearranged horizontally or vertically. So the image can be treated as a vector (called feature vector).

### 2.7. Calculation Total Confidence Value

In the present work, wavelet transform is used on the elliptic shape face region once which divide the whole image into 4 equal sized subimages, namely, low-frequency LL band (approximate component) and three high-frequency bands (detailed components), HL, LH, and HH. Then the pixelwise average of the detail components is computed using

(5) $$\begin{array}{c} D\left(x,y\right)=\frac{1}{3}\left(A\left(x,y\right)+B\left(x,y\right)+C\left(x,y\right)\right), \end{array}$$

where  *A*(*x*, *y*) is the HL band subimage, *B*(*x*, *y*) is the LH band sub-image and *C*(*x*, *y*) is the HH band subimage. *D*(*x*, *y*) is the average subimage of *A*(*x*, *y*), *B*(*x*, *y*), and *C*(*x*, *y*) band subimages, and *x*, *y* are spatial coordinates.

**Figure 9:**
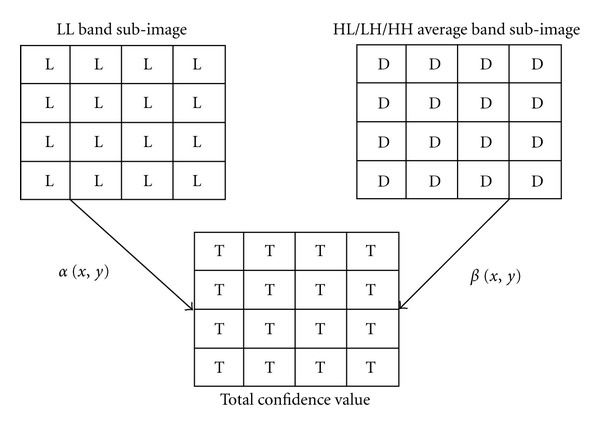
Mixing technique.

Next, a matrix called total confidence matrix *T*(*x*, *y*) is formed by taking a pixelwise weighted sum of pixel values of LL band and average subimages [19–21] using (6), as given in the following:

(6) $$\begin{array}{c} T\left(x,y\right)=\left(\alpha \left(x,y\right)\times L\left(x,y\right)\right)+\left(\beta \left(x,y\right)\times D\left(x,y\right)\right), \end{array}$$

where *T*(*x*, *y*) is the total confidence value, *L*(*x*, *y*) is the LL band subimages, and *D*(*x*, *y*) is the average of HL/LH/HH band subimages, while *α*(*x*, *y*) and *β*(*x*, *y*) denote the weighting factors for pixel values of LL band and HL/LH/HH average band subimages, respectively, which are shown in Figure 9.

After calculating the total confidence matrices for all the images, each matrix is transformed into a horizontal vector, by concatenating the rows of elements in it. This process is repeated for all the images in the database. Let the number of elements in each such horizontal vector be *N*, where *N* is the product of the number of rows and columns in LL band or average subimages. By placing the horizontal vectors in row order, a new matrix of size *M* × *N* is formed, where *M* is the number of images in the database. Thus, *M* × *N* matrix is divided into two parts by the size of (*M*/2) × *N*, of which one part will be used for training purpose and the other part for testing purpose only. The first part contains odd number of images like first row, third row, fifth row, and so on from *M* × *N* matrix, and the second part contains even number of images like second row, fourth row, sixth row, and so on from *M* × *N* matrix.

### 2.8. Eigenface for Recognition

Principal component analysis (PCA) [22, 23] is performed on training set described above which gives a set of eigenvalues and corresponding eigenvector. Each eigenvector can be shown as sort of ghostly face which is called an eigenface. Each face image in the training set can be represented exactly in terms of a linear combination of these eigenfaces. So the number of rows that is, number of face images in the training set, is equal to the number of a eigenfaces. However, the faces can also be approximated using only the “best” eigenfaces, those that have the largest eigenvalues and which therefore account for the most variance within the set of face images. For this, the eigenvalues are sorted in descending order and eigenvectors corresponding to a few largest eigenvalues are retained. The *n*-dimensional space that is formed by these eigenvectors or eigenfaces is called eigenspace. The face images in the training set are then projected onto the eigenspace to get the corresponding eigenfaces, which are then used to train a classifier. For the test face images, similar procedure is followed to get their corresponding eigenfaces, which are classified by the trained classifier.

### 2.9. Local Binary Pattern

**Figure 10:**
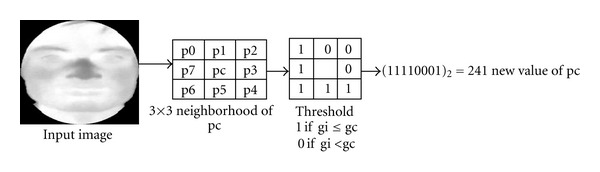
Local binary pattern.

The second method of feature is local binary pattern (LBP). The LBP is a type of feature used for texture classification in computer vision. LBP was first described in 1994 [24, 25]. It has since been found to be a powerful feature for texture classification. As it can be appreciated in Figure 10, the original LBP operator represents each pixel of an image by thresholding its 3 × 3 neighborhood with the center value and considering the result as a binary number, called the LBP code. In the classification step, the image is usually divided into rectangular regions and histograms of the LBP that codes are calculated over each of them. The histograms of each region are concatenated into a single one, and a dissimilarity measure is used to compare the histograms of two different images.

### 2.10. Multilayer Feed Forward Neural Network

Artificial neural networks (ANNs) [26, 27] possess extraordinary generalization capability to obtain useful information from complex environment or data. So ANN can be used to extract patterns and detect trends that are too hard to be found by either humans or other computer techniques. A trained ANN can be thought of as an “expert system.” The back propagation learning algorithm is one of the most popular neural networks to the scientific and engineering community for modeling and processing of many quantitative phenomena. This learning algorithm is applied to multilayer feed forward networks consisting of processing elements with continuous differentiable activation functions. The five layer feed forward back propagation neural network is used here as a classifier. Momentum allows the network to respond to local gradient and to recent trends in the error surface. The momentum is used to back propagation learning algorithm for making weight changes equal to the sum of a fraction of the last weight change and the new change. The magnitude of the effect that the last weight change is allowed is known as momentum constant (mc). The momentum constant may be any number between 0 and 1. The momentum constant zero means, a weight changes according to the gradient and the momentum constant one means, the new weight change is set to equal the last weight change, and the gradient is not considered here. The gradient is computed by summing the gradients calculated at each training example, and the weights and biases are only updated after all training examples have been presented. Tan-sigmoid transfer functions are used to calculate a layer's output from its net, the first input, and the next three hidden layers and the outer most layer gradient descent with momentum training function is used to update weight and bias values.

### 2.11. Minimum Distance Classifier

Recognition techniques based on matching represent each class by a prototype pattern vector. It places an unknown pattern in the class to which it is closest in terms of a predefined metric. The simplest approach is the minimum distance classifier [15]. It must determine the Euclidean distance between the unknown pattern and each of the prototype vectors. It chooses the smallest distance to take a judgment. The prototype of each pattern class is represented as the mean vector of the patterns of that class which is expressed using

(7) $$\begin{array}{c} m_{j}=\frac{1}{N_{j}}\sum_{x\in \omega_{j}}x_{j},\;j=1,2,3,\ldots ,W, \end{array}$$

where *W* is the number of pattern classes, *ω*_*j*_ is the set of pattern vectors of class *j*, and *N*_*j*_ is the number of pattern vectors in *ω*_*j*_. In order to get the class membership of an unknown pattern vector *x*, its closest prototype is searched using Euclidean distance measure, which is shown in

(8) $$\begin{array}{c} D_{j}\left(x\right)=||x-m_{j}||,\;j=1,2,3,\ldots ,W. \end{array}$$

If *D*_*j*_(*x*) is the smallest distance, that is, best match, then assign *x* to class *ω*_*j*_.

## 3. Experiment and Results

Experiments have been performed on our own captured thermal face images at our laboratory and Terravic Facial Infrared database. In our Database, there are 17 × 34 = 578 thermal images. The details of our database have been mentioned in Section 2.1. Twelve images are taken in each person for our experiments from two above-mentioned datasets, out of which 6 face images are used to form training set and 6 face images are used to form testing set. We have made all the images of size 112 × 92. The Terravic Facial Infrared Database contains total number of 20 classes (19 men and 1 woman) of 8-bit gray scale JPEG thermal faces of 320 × 240. Size of the database is 298 MB, and images with different rotations are left, right, and frontal face images also available with different items like glass and hat [13]. Experimental process can be divided into several ways.

### 3.1. Harr Wavelet + PCA + ANN

In the first set of experiments, Haar wavelet is used to decompose the cropped face image once which produces 4 subimages as LL, HL, LH, and HH bands. Then the average of HL/LH/HH band subimages is computed using (5). We have used ten different sets of values for (*α*, *β*) to generate 10 different confidence matrices for each face image. The values of *α* and *β* are chosen according to

(9) β=0.1×i,α=1.0−β,0≤i≤10.

**Figure 11:**
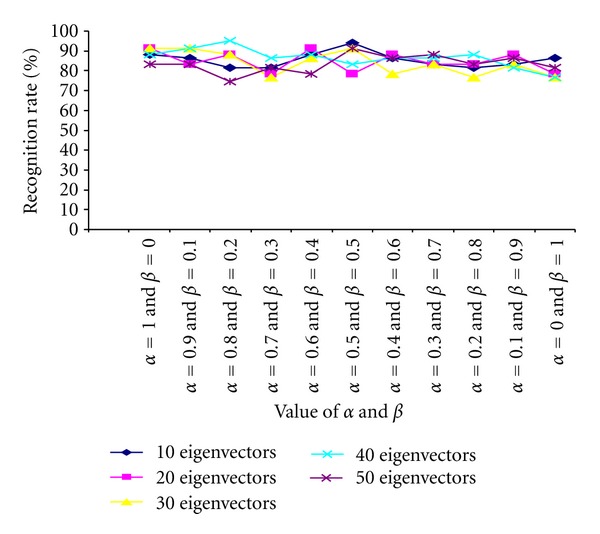
Comparative study of recognition performance (own database) with varying numbers of eigenvectors and values of *α* and *β*.

**Figure 12:**
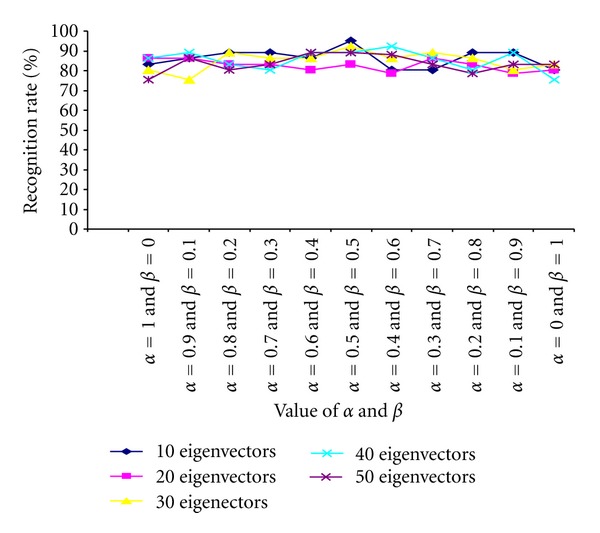
Comparative study of recognition rate (performed on Terrivic Facial Thermal Database) with varying numbers of eigenvectors and values of *α* and *β*.

After computing the confidence matrices of all the decomposed face images, PCA is performed on these confidence matrices for further dimensionality reduction. ANN classifier (with 0.02 acceleration and 0.9 momentum) is then used to classify the face images on the basis of the extracted features. The recognition performances of the classifier on our own Database and Terravic Facial IR database are shown in Tables 3 and 4, respectively. The results are also shown graphically in Figures 11 and 12, respectively.

### 3.2. Harr Wavelet + PCA + Minimum Distance Classifier

**Figure 13:**
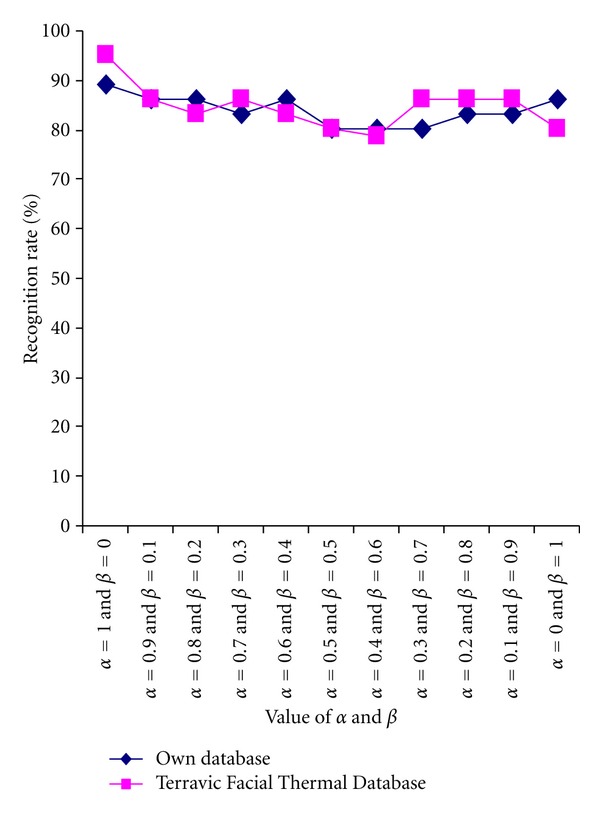
Comparative study of Recognition performance (own database and benchmark database) with minimum distance classifier and the values of *α* and *β*.

In the second set of experiments, the feature set was kept the same as those in the first set of experiments, but the classifier is chosen as minimum distance classifier. The recognition performance obtained on both the thermal face databases considered here is detailed in Table 5 and also graphically compared in Figure 13. 

### 3.3. Local Binary Pattern + (PCA + ANN/Minimum Distance Classifier)

**Figure 14:**
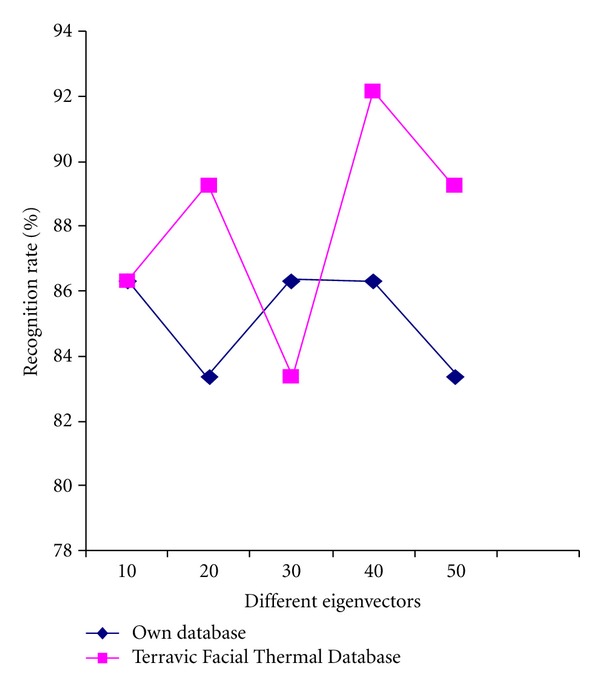
Recognition performance (own database and benchmark database) with varying numbers of eigenvectors, ANN and minimum distance classifier.

In the third set of experiments, cropped face images are divided in to 161 subimages each of size 8 × 8 pixels. Then local binary pattern is used to extract features from each of the subimages which are concatenated in row wise manner. After performing PCA on the LBP features for dimensionality reduction, ANN and minimum distance classifier are used separately for recognition of the face images on the basis of the extracted features. The obtained recognition results are shown in Table 6. The results are also shown graphically in Figure 14. 

## 4. Conclusions

In this paper, a comparative study of thermal face recognition methods is discussed and implemented. In this study two local-matching techniques, one based on Haar wavelet and the other based on Local Binary Pattern, are analyzed. Firstly, human thermal face images are preprocessed and cropped the face region only, from the entire face images. Then above-mentioned two feature extraction methods are used to extract features from the cropped images. Then, PCA is performed on the individual feature set for dimensionality reducation. Finally, two different classifiers are used to classify face images. One such classifier is multilayer feed forward neural network, and another is minimum distance classifier. The experiments have been performed on the database created at our own laboratory and Terravic Facial IR Database. The proposed system gave higher recognition performance in the experiments, and the recognition rate was 95.09% for *α* = 0.8, *β* = 0.2, and number of eigenvectors is 40. This experiment was performed on our own database, which is shown in Table 3. Furthermore, no knowledge of geometry or specific feature of the face is required. However, this system is applicable to front views and constant background only. It may fail in unconstraint environments like natural scenes.

## Figures and Tables

**Algorithm 1:**
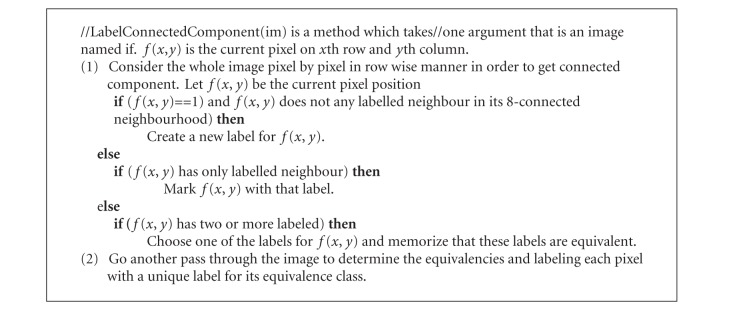
LabelConnectedComponent(im).

**Algorithm 2:**
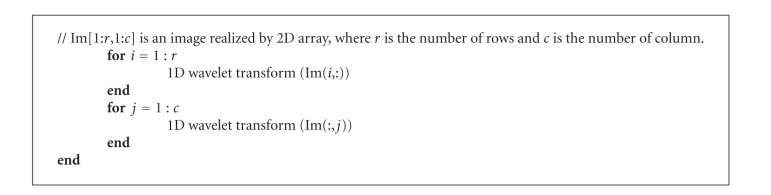
Function StandardDecomposition(Im[1:*r*,1:*c*]).

**Table 1:** Wavelength ranges for different infrared spectrums.

Spectrum	Wavelength range
Visible spectrum	0.4–0.7 *μ*m (micrometer/micron)
Near infrared (NIR)	0.7–1.0 *μ*m (micrometer/micron)
Short-wave infrared (SWIR)	1–3 *μ*m (micrometer/micron)
Mid-wave infrared (MWIR)	3–5 *μ*m (micrometer/micron)
Thermal infrared (TIR)	8–14 *μ*m (micrometer/micron)

**Table 2:** Resolution, mean, and the detail coefficients of full decomposition.

Resolution	Mean	Detail coefficients
4	[10 4 9 5]	
2	[7 7]	[3 2]
1	[7]	[0]

**Table 3:** Recognition performance (own database) with varying numbers of eigenvectors and the values of *α* and *β*.

Value of *α* and *β*	Recognition rate (%)				
	10 eigenvectors	20 eigenvectors	30 eigenvectors	40 eigenvectors	50 eigenvectors
*α* = 1.0 and *β* = 0.0	88.23	91.18	91.18	88.23	83.33
*α* = 0.9 and *β* = 0.1	86.27	83.33	91.18	91.18	83.33
*α* = 0.8 and *β* = 0.2	81.38	88.23	88.23	95.09	74.50
*α* = 0.7 and *β* = 0.3	81.38	78.57	76.74	86.27	81.38
*α* = 0.6 and *β* = 0.4	88.23	91.18	86.27	88.23	78.57
*α* = 0.5 and *β* = 0.5	94.11	78.57	91.18	83.33	91.18
*α* = 0.4 and *β* = 0.6	86.27	88.23	78.57	86.27	86.27
*α* = 0.3 and *β* = 0.7	83.33	83.33	83.33	86.27	88.23
*α* = 0.2 and *β* = 0.8	81.38	83.33	76.74	88.23	83.33
*α* = 0.1 and *β* = 0.9	83.33	88.23	83.33	81.38	86.27
*α* = 0.0 and *β* = 1.0	86.27	78.57	76.74	76.74	81.38

**Table 4:** Recognition performance (benchmark database) with varying numbers of eigenvectors and the values of *α* and *β*.

Value of *α* and *β*	Recognition rate (%)				
	10 eigenvectors	20 eigenvectors	30 eigenvectors	40 eigenvectors	50 eigenvectors
*α* = 1.0 and *β* = 0.0	83.33	86.27	80.39	86.27	75.49
*α* = 0.9 and *β* = 0.1	86.27	86.27	75.49	89.22	86.27
*α* = 0.8 and *β* = 0.2	89.22	83.33	89.22	83.33	80.39
*α* = 0.7 and *β* = 0.3	89.22	83.33	86.27	80.39	83.33
*α* = 0.6 and *β* = 0.4	86.27	80.39	86.27	89.22	89.22
*α* = 0.5 and *β* = 0.5	94.11	83.33	92.15	89.22	89.22
*α* = 0.4 and *β* = 0.6	80.39	78.57	86.27	92.15	88.22
*α* = 0.3 and *β* = 0.7	80.39	86.27	89.22	86.27	83.33
*α* = 0.2 and *β* = 0.8	89.22	83.33	86.27	80.39	78.57
*α* = 0.1 and *β* = 0.9	89.22	78.57	80.39	89.22	83.33
*α* = 0.0 and *β* = 1.0	80.39	80.39	83.33	75.49	83.33

**Table 5:** Recognition performance (on own database and benchmark database) with minimum distance classifier and the value of *α* and *β*.

Value of *α* and *β*	Recognition rate (%)	
	Own database	Terravic Facial
		Thermal Database
*α* = 1.0 and *β* = 0.0	89.22	94.11
*α* = 0.9 and *β* = 0.1	86.27	86.27
*α* = 0.8 and *β* = 0.2	86.27	83.33
*α* = 0.7 and *β* = 0.3	83.33	86.27
*α* = 0.6 and *β* = 0.4	86.27	83.33
*α* = 0.5 and *β* = 0.5	80.39	80.39
*α* = 0.4 and *β* = 0.6	80.39	78.57
*α* = 0.3 and *β* = 0.7	80.39	86.27
*α* = 0.2 and *β* = 0.8	83.33	86.27
*α* = 0.1 and *β* = 0.9	83.33	86.27
*α* = 0.0 and *β* = 1.0	86.27	80.39

**Table 6:** Recognition performance (own database and benchmark database) with varying numbers of eigenvectors, ANN, and minimum distance classifier.

	Recognition rate (%)					
	ANN classifier					Minimum distance classifier
	10 eigenvectors	20 eigenvectors	30 eigenvectors	40 eigenvectors	50 eigenvectors
Own database	86.27	83.33	86.27	86.27	83.33	89.22
Terravic Facial Thermal Database	86.27	89.22	83.33	92.15	89.22	94.11
